# Collagen in Wound Healing

**DOI:** 10.3390/bioengineering8050063

**Published:** 2021-05-11

**Authors:** Shomita S. Mathew-Steiner, Sashwati Roy, Chandan K. Sen

**Affiliations:** Indiana Center for Regenerative Medicine and Engineering, School of Medicine, Indiana University, Indianapolis, IN 46202, USA; sssteine@iu.edu (S.S.M.-S.); roysa@iu.edu (S.R.)

**Keywords:** extracellular matrix, collagen, signaling, inflammation, wound healing, collagen dressings, engineered collagen

## Abstract

Normal wound healing progresses through inflammatory, proliferative and remodeling phases in response to tissue injury. Collagen, a key component of the extracellular matrix, plays critical roles in the regulation of the phases of wound healing either in its native, fibrillar conformation or as soluble components in the wound milieu. Impairments in any of these phases stall the wound in a chronic, non-healing state that typically requires some form of intervention to guide the process back to completion. Key factors in the hostile environment of a chronic wound are persistent inflammation, increased destruction of ECM components caused by elevated metalloproteinases and other enzymes and improper activation of soluble mediators of the wound healing process. Collagen, being central in the regulation of several of these processes, has been utilized as an adjunct wound therapy to promote healing. In this work the significance of collagen in different biological processes relevant to wound healing are reviewed and a summary of the current literature on the use of collagen-based products in wound care is provided.

## 1. Introduction

**Figure 1:**
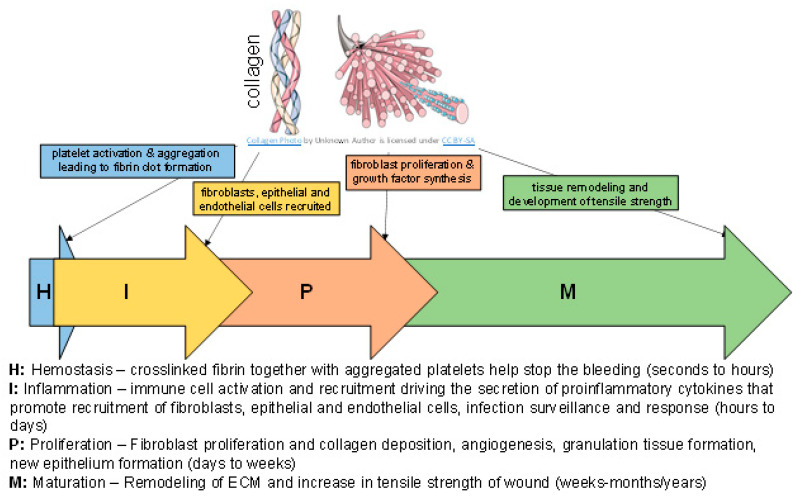
Brief summary of wound healing phases.

Sophisticated regulation by a number of key factors including the environment of the wound which is rich in extracellular matrix (ECM) drives the process of wound healing [1,2]. The complex macromolecules constituting the ECM include fibrous components (e.g., collagens and elastins) and glycoprotein components (e.g., fibronectin, proteoglycans and laminins). Each of these molecules interact to drive the process of tissue function, growth and repair [3,4,5]. Wound repair is a complex process that is broadly categorized into the following four phases which occur in a temporal sequence but are overlapping: hemostasis, inflammation, proliferation (cellular infiltration, angiogenesis and re-epithelialization) and maturation/remodeling (Figure 1) [1]. Key steps of the wound healing process, such as hemostasis, inflammation and angiogenesis are responsive to the ECM, collagen and its compounds [1,6,7,8,9,10,11,12,13]. In response to injury, collagen induces platelet activation and aggregation resulting in the deposition of a fibrin clot at the injury site. In the inflammatory stage of wound healing, immune cell activation drives the secretion of proinflammatory cytokines which influence migration of fibroblasts, epithelial and endothelial cells. Fibroblasts contribute to collagen deposition. Simultaneously, collagen degradation releases fragments that promote fibroblast proliferation and synthesis of growth factors that lead to angiogenesis and re-epithelialization. Finally, the remodeling of the ECM (balance of new matrix synthesis and matrix metalloproteinase degradative activities) determines the acquisition of tensile strength [14,15,16]. In this work we sought to briefly review the significance of collagen in different biological processes relevant to wound healing. The current literature on the use of collagen-based products in wound care is summarized.

## 2. Types of Collagens in the Skin and Wound

Collagens are the most abundant protein found throughout the body. In the healing wound, these collagens are synthesized by cells such as fibroblasts and modified into complex morphologies [17,18,19,20,21]. The type, amount and organization of collagen changes in the healing wound and determines the tensile strength of the healed skin. Collagen III is the first to be synthesized in the early stages of wound healing and is replaced by collagen I, the dominant skin collagen. The initial random deposition of collagen during the granulation tissue formation is further enhanced by lysyl oxidase enzyme-induced covalent cross-linking. This process matures the collagen into complex structures that are reoriented for tensile strength restoration. Collagen remodeling continues for months after wound closure and the tensile strength of the repaired tissue increases to about 80–85% of normal tissue if all processes proceed without any perturbations [16].

In the skin, the fibrillar collagens types I, III and V are the most common, followed by fibril-associated collagens type XII, XIV, XVI, and VI. The non-fibrillar collagens type IV, XVIII are found in the basement membrane of the skin [14,18,19,22,23].

## 3. Processing of Collagen in the Skin and Wound

### 3.1. Biosynthesis and Cross-Linking

In the healing wound, cells such as fibroblasts (resident, and myeloid cell converted fibroblasts) [24] are the main source of newly synthesized collagen. The biosynthesis activities of fibril-forming collagens are the most extensively studied among all the collagens and involve multiple complex steps requiring the temporal and spatial coordination of several biochemical events [21,25]. Following transcription, the nascent/pre-pro-collagen is post-translationally modified in the endoplasmic reticulum into pro-collagen with the removal of the signal peptide on the N-terminus. Hydroxylation and glycosylation of amino acid residues results in the formation of the triple-helical structure characteristic of collagens. Supported by chaperone proteins, the pro-collagen triple-helical structure is stabilized for further processing and maturation in the Golgi apparatus and assembled into secretory vesicles that are extruded into the extracellular space where the pro-collagen is enzymatically modified into tropocollagen. The final collagen fibril assembly occurs by covalent cross-linking. The mechanical properties (elasticity and reversible deformation) of fibrillar collagens are dependent on this cross-linking process. Some of these cross-links include: (1) disulfide bonds; (2) reducible and mature cross-links produced via the lysyl oxidase pathway; (3) transglutaminase cross-links; and (4) advanced glycation end (AGE) product-mediated cross-links, among others. The nuances of cross-linking vary with the type of collagen and the tissue context and creates a multi-layered hierarchical structure [26]. Mature cross-links add resistance to shear stress. AGE-specific cross-links contribute to increased stiffness of collagens in aged tissues.

### 3.2. Degradation

Collagen degradation is involved in inflammation, angiogenesis, and re-epithelialization in the wound regulated by complex molecular pathways [27]. During the inflammation phase, soluble fragments from collagen degradation recruit immune cells such as macrophages that patrol the wound for removal of microbes and devitalized tissue. This aids in the transition to the proliferative phase. During this stage, collagen fragments serve as potent angiogenic signals to promote the development of new blood vessels. Keratinocyte migration is also promoted by collagen and contributes to wound re-epithelialization [16,28,29]. Degradation is regulated by extracellular and intracellular pathways. The former involves membrane-bound and secreted proteolytic enzymes. The latter involves internalization of intact collagen fibrils and fragmented collagen (through phagocytosis, macropinocytosis or endocytosis), followed by enzymatic breakdown. Defects in the regulated turnover of collagens results in pathological conditions such as fibrosis [20,30,31,32].

The actions of proteolytic enzymes at different stages in the wound healing process guides the remodeling of the repaired tissue. Two important enzyme families are the matrix metalloproteinases (MMPs) and serine proteases. The production and secretion of these enzymes are tightly regulated and are associated with specific cellular subtypes [12,33]. Among the MMPs, collagenases and gelatinases, which degrade intact and damaged fibrillar collagen respectively, are key for collagen turnover during wound healing. Collagens I and III are preferentially cleaved by MMP-1 (also called collagenase-1) and MMP-8 (collagenase-2) while collagen IV is degraded by the gelatinase MMP-9. Extensive research has determined that collagenolytic enzymes can recognize, bind, unwind and cleave the individual strands of the triple helix. It is speculated that this high specificity could be driven by the primary and super-secondary structures of collagen. MMPs drive physiological (development and tissue repair) and pathological (tumorigenesis and metastasis) processes. They also contribute to the release of bioactive fragments (also termed matricryptins) such as endostatin and tumstatin from full-length collagens [34]. These fragments specifically guide blood vessel pruning that in turn enables the re-establishment of the tissue architecture during healing [23,35,36,37,38]. Neutrophil elastase is a serine protease that aids in the same process. A balance of enzyme activity and inhibition is required for normal wound healing and is under tight regulatory control. Imbalances in the levels of these enzymes are a factor in wound chronicity. Wounds infected with microbes that produce these collagen-degrading enzymes add to the imbalance, leading to chronic wounds.

### 3.3. Receptor-Mediated Signaling

Collagen in all its forms, triple-helical, matrix-incorporated and degraded fragments, are cognate ligands of diverse families of cell surface receptors including integrins, receptor tyrosine kinases and immunoglobulin type receptors [1,39]. In the wound environment, collagens mediate several key steps such as platelet aggregation, inflammation modulation, angiogenesis, granulation tissue formation and re-epithelialization in a integrin signaling-dependent manner [31,32,39]. Receptor tyrosine kinases such as Discoidin Domain Receptors (DDR-1 and DDR-2) bind matrix-incorporated collagen and regulate key wound healing processes. Loss of function of these signaling molecules inhibits keratinocyte proliferation and collagen remodeling during wound healing, resulting in wounds with low tensile strength [40]. Abnormal signaling induced by collagen is observed in pathological conditions such as scar formation [32].

## 4. Roles for Collagen in the Skin and Wound

Collagen contributes to the mechanical strength and elasticity of tissues and acts as a natural substrate for cellular attachment, proliferation, and differentiation (Figure 1) [16,21,28,29]. Biofilm-mediated upregulation of MMP-2 via microRNAs creates a collagenolytic environment in the wound, sharply decreasing the collagen I/collagen III ratio and compromising the biomechanical properties of the repaired skin, possibly making the repaired skin vulnerable to wound recurrence [41]. A recent mapping study of collagen structure and function suggested that in normal, injured tissue the collagen fibril is in a closed conformation that upon exposure to blood following injury exposes cell- and ligand-binding sites that could promote the wound healing process [20]. Several recent reviews detail roles of collagen in the skin and wounds [1,42,43,44,45,46,47,48,49,50].

### 4.1. Role in Inflammation

The inflammatory phase of wound healing includes hemostasis and inflammation [51]. Collagen exposure due to injury activates the clotting cascade, resulting in a fibrin clot that stops the initial bleeding. Collagen I and IV fragments can be mediators of inflammation by acting as potent chemoattractants for neutrophils, enhancing phagocytosis and immune responses and modulating gene expression [19,34]. Inflammation is a critical step in the normal process of wound healing and drives the proliferation of fibroblasts which synthesize collagen and ECM [9]. The resolution of inflammation in a timely manner is equally important in normal wound healing. Resolution of inflammation is an active process that is driven by balanced pro and anti-inflammatory responses. A study using a stabilized collagen matrix showed that collagen mounts a robust and sharp inflammatory response that is transient and resolves rapidly to make way for wound healing to advance [6]. Furthermore, an important role for collagen in promoting an anti-inflammatory, pro-angiogenic wound macrophage phenotype via microRNA signaling has also been demonstrated [7,10].

### 4.2. Role in Angiogenesis

Angiogenesis, a critical component of physiological (development, wound healing) and pathological (cancer) processes, is tightly regulated by the balanced activity of stimulators and inhibitors. ECM remodeling provides critical support for vascular development and collagens play an important role in this process [7,11,13,52,53]. Depending on the type of collagen, the role might be as a promoter or inhibitor of angiogenesis. A live analysis via multiphoton microscopy of neovessel formation in vitro identified a dynamic modulation of collagen I that showed early stage remodeling of collagen fibrils progressing to collagen condensation in later stages of development [54]. Collagen I is known to potently stimulate angiogenesis in vitro and in vivo through engagement of specific integrin receptors. Specifically, the C-propeptide fragment of collagen I recruits endothelial cells, potentially triggering angiogenesis in the healing wound [12]. By contrast, proteolytic collagen fragments of collagen IV and XVIII (e.g., endostatin, arresten, canstatin, tumstatin) show anti-angiogenic properties [23,35]. Studies have shown a role for these fragments in inhibiting proliferation and migration of endothelial cells and inducing endothelial cell apoptosis. These fragments are of interest in curbing angiogenesis in several pathological conditions [12,19,34].

### 4.3. Role in ECM Remodeling

Collagens are a structural component of the ECM that contribute to skin flexibility in addition to stabilizing growth factors and regulating cell adhesion and signaling between cells and ECM. In the process of wound healing, as the wound tissue undergoes remodeling over years, the adult wound heals with the formation of a ‘normal’ scar. The scar tissue regains anywhere from 50–80% of the original tensile strength of normal skin but may be functionally deficient [55]. The main difference between the scar and unwounded skin appears to be the density, fiber size and orientation of the collagen fibrils [28].

Abnormalities in the ECM reconstitution during wound healing result in hypertrophic and keloid scars. Scarring is a consequence of altered levels of the same molecules that typically make up the ECM^,^ i.e., collagen I and III, fibronectin and laminin are abnormally high in scar tissue [55]. Collagen fiber orientation in scars (normotrophic, hypertrophic and keloid) are parallel to the epithelial surface unlike that of normal skin where the fibers form a three-dimensional basketweave-like network [56]. There are structural and compositional differences between these types of scars. Keloid scars are characterized by abnormally thick bundles of collagen that are poorly organized with fewer cross-links that are found in the deep dermis compared to superficial dermic. Hypertrophic scars have thinner collagen bundles than keloid or normotrophic scars [57,58,59]. The ratio of collagen I to III is higher in keloids than normotrophic scars. Even within the keloid scar, there is a heterogeneity to the collagen I/III ratio [14].

## 5. Effect of Aging on Collagen in the Skin and Wound

The aged skin has lower collagen density that is increasingly cross-linked and fragmented [60,61]. Together with senescence, collagen fiber remodeling results in increased stiffness. Furthermore, the aging skin has a higher percentage of collagen III [62]. Collagen organization visualized through Fourier transformed infrared imaging, scanning electron microscopy and histological staining showed fragmented, clustered and coarse fiber bundles that are oriented parallel to the skin surface in aging skin [63,64,65]. Age-induced alterations (reduced collagen deposition and increased non-enzymatic cross-linking) in collagen impact the mechanical environment of the skin and predispose it to wound healing impairments [66,67,68].

## 6. Collagen Formats and Applications in Wound Healing

**Figure 2:**
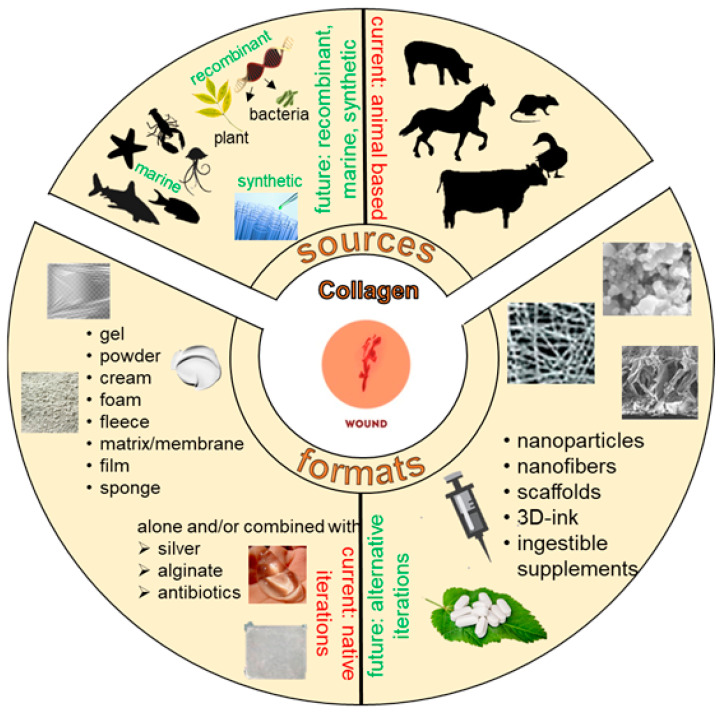
Sources and formats of collagen for wound healing applications.

Aberrations in the normal progression through the wound healing phases results in the development of chronic wounds that need to be managed appropriately for healing to complete. Key factors in the hostile environment of a chronic wound are persistent inflammation, increased destruction of ECM components due to elevated MMPs and improper activation of soluble mediators of the wound healing process. Because collagen is an important regulator of several of these processes, it has been utilized as an adjunct wound therapy to promote healing. Biocompatibility, low immunogenicity, ability to recruit wound healing responsive cells (macrophages, fibroblasts etc.) and ease of application are some of the reasons why collagen-based biomaterials have been used for wound dressings. Standard collagen sources are typically bovine, equine, avian or porcine in origin (Figure 2) [69]. There are significant disadvantages associated with the use of animal-based collagen products including development of allergic reactions, transmission of prion diseases (e.g., bovine spongiform encephalopathy) and microbial contamination [70,71]. Furthermore, in some communities there are religious constraints associated with use of bovine- and porcine-derived tissue. Therefore, alternative natural (marine) or engineered (recombinant human collagen from bacterial or plant material) sources of collagen have been considered.

Collagen applied as adjunct therapy in wound healing could promote healing potentially by acting as: (i) a decoy/sink for the raging MMPs and other enzymes in the wound thereby abating inflammation and restoring progression into the reparative stages; (ii) a substrate aiding in the migration of key cellular components of wound healing; or (iii) a promoter of a proangiogenic, anti-inflammatory environment to resolve the injury towards healing [6,7,8,10,17,53].

### 6.1. Collagen Wound Dressings

Collagen applications in wound healing have been tested in numerous ways. They have been used as matrices/scaffolds for tissue engineering, hemostatics, soft tissue repair and more recently as a drug delivery system [6,10,11,26,53,72,73,74,75,76,77,78,79,80]. Collagen wound dressings contain collagen blended with natural and synthetic polymers such as polyethylene oxide, poly (L-lactic acid), hyaluronic acid, elastin and silk fibroin, alginate, chitosan, etc [73,81,82,83,84,85]. These blended fabrications have incorporated other additives such as insulin [86], antibiotics [87,88,89,90,91,92] or gold nanoparticles [93,94,95] and have been tested mostly in in vitro studies or small animal models of wound healing. An evidence-based review of clinical studies on antibacterial integrated collagen wound dressings indicated that most studies were limited by small sample sizes and mixed chronic wound etiologies [46]. Therefore, although positive outcomes are reported, robust evaluation of the specific value of these integrated wound dressings as it related to clinical diabetic foot ulcers remains promising but inconclusive. The need for larger, standardized clinical studies to claim treatment efficacy thus arises.

A plethora of formulations of collagen as amorphous gels, sheet or powder forms, combined with other agents (e.g., silver, for the antimicrobial properties, or ethylenediaminetetraacetic acid (EDTA), carboxymethyl cellulose (CMC) or alginate, i.e., collagen enhancers) are available as wound dressings in the market (Figure 2) [6,7,8,10,46,53,77,96]. The sponge/fleece version of collagen has been tested as a cell-free matrix that promoted new tissue formation in a limited study [47,75]. Particulate or powdered collagen have minimal covalent cross-linking and are active upon administration as signaling molecules [77].

Collagen is also used as a surface coating to enhance moisture retention and promote cell adhesion within scaffolds/matrices [73,81,82,83,84,86,87,88,89,90,91,92,93,94,95]. Water retention is important to keep the wound bed moist. The arginine-glycine-aspartic acid (RGD) sites on collagen binds integrins of cells and promotes cell adhesion and migration of fibroblasts and keratinocytes in in vitro studies. A collagen-coated scaffold implanted in a rat burn wound model showed faster wound re-epithelialization and healing compared to standard of care dressing [97].

The technological processing of collagen tissues to hydrolysates and subsequent rebuilding into formats used for clinical applications was recently reviewed [98]. Several publications show the potential for collagen as a flexible biomaterial for wound healing. However, there is still a need for high-quality studies and randomized control trials to support their clinical applications [46,75].

In recent years interest in collagen nanostructures has surged [78]. Nano collagen is a relatively new material and is made of collagen reduced to a nanoparticulate size. This nano-size provides a higher surface area-to-volume ratio. Electrospinning is the primary technique used to produce biocompatible nano collagen fibers. Collagen nanoparticles have been tested for their application as therapeutic drug delivery systems. For example, a gold-loaded hydroxyapatite collagen nanostructure was tested in vitro and was shown to promote cell adhesion, growth and proliferation [99]. The localized delivery of therapeutic factors using a material that is stable and compatible with the tissue microenvironment of the wound is a key advantage of nano collagens [78]. Insufficient knowledge and research of these nano particles is a limiting factor and requires additional in-depth study.

#### 6.1.1. Recombinant Human Collagen

The risks associated with the use of animal sources of collagen can be overcome by the use of alternatively sourced collagen. Plant-derived human collagen (PDHC; typically recombinant human collagen engineered in plants like tobacco) have similar scaffold properties to wild-type human collagen [100,101,102,103,104]. Various formulations (gel, matrix, electrospun scaffolds and lyophilized sponges) of PDHC have been experimentally derived and tested (Figure 2) [102]. A recent pilot study used PDHC on chronic ulcers of various etiologies and demonstrated that the product was safe for use and promoted faster wound closure [105].

A potential problem with recombinant human collagens from non-animal sources is the requirement for post-translational proline hydroxylation that potentially limits large scale production [106]. The discovery of collagen-like proteins, Scl1 and Scl2 from *Streptococcus pyogenes* led to the generation of constructs in a recombinant E. coli system in an effort to establish large-scale production methods. Bacterial-derived collagens serve as a biosynthetic ground-up approach, where non-animal collagen with no specific bioactivity can be manipulated for desired interactions. A test of this system in the context of human mesenchymal stem cells was shown to have chondrogenic potential [107].

#### 6.1.2. Marine Collagen

Collagen I has been extracted from various marine sources such as fish skin, jellyfish, sponges and squid (Figure 2) [70,71,108,109,110,111,112,113,114,115,116]. Marine-derived collagen I was shown to promote wound healing in experimental (rodent models) and clinical studies [117,118,119]. The marine source of collagen is beneficial because the abundance of material that would typically be considered ‘waste products’ in the fish processing industry can be recycled into collagen-based wound dressings as well as derivatized into dietary supplements for weight management and sugar control [108].

Marine-derived collagens are chemically and mechanically different from mammalian collagen but are considered favorable for biomedical applications due to high biodegradability and biocompatibility and low immunogenicity [70]. Marine-derived collagen I thermostability has been tested by several groups and found to be lower than mammalian-derived collagen suggesting a need for engineering additional cross-links prior to use in wound healing or other biological applications. These differences have been attributed to the amino acid (particularly glycine, proline and hydroxyproline) content of the collagen I [69]. A systematic review of collagen from marine sources in skin wound healing described studies performed in animal models and highlighted the potential for wound healing applications [70]. The field is now ready for clinical trials. 

### 6.2. Percutaneous Collagen Induction

In 1995, a method called subcision was introduced as a minimally invasive way to treat scars using extremely fine needles to disrupt dermal collagen to trigger dermal remodeling and skin resurfacing [120,121,122]. This method is now known as microneedling or percutaneous collagen induction (PCI). The microneedles puncture the outer layers of the skin into the papillary dermis to initiate the release of growth factors that trigger collagen I and elastin formation. This method has been applied to the treatment of acne, scars, facial rejuvenation, alopecia, pigmentation issues, etc. in small cohort clinical studies. A recent review of the evidence available on PCI techniques for scar treatment identified benefits such as little to no side effects and versatility of application [120]. However, this and other reviews have pointed out the lack of high-quality studies with sufficient numbers of patients following a standardized outcome protocol [121,122].

### 6.3. Hydrolyzed Collagen

Native collagen can be denatured and hydrolyzed with acids, alkali or thermal treatment (with enzymatic digestion) to produce low molecular weight (3–6 KDa) peptides with unique physicochemical and biological properties compared to the native form [123]. The advantages of hydrolyzed collagen (HC) are that it is highly soluble, easily absorbed and distributed in the human body, cost effective, easily emulsified and stabilized for use. However, a disadvantage is that unlike the native form, HC needs to be combined with other biopolymers (cellulose or chitosan) to form scaffolds or films. Interestingly, HC has antioxidant and antimicrobial activities. Hydrogel preparations of HC were shown to have antibacterial activity against *Escherichia coli* and *Staphylococcus aureus*. These preparations were also shown to promote cell proliferation and migration and burn wound healing. HC in electrospun nanofibrous scaffolds was shown to have biomechanical and antimicrobial properties [81,110]. A recent review on the different types of HC, sources and applications as biomaterials provides additional details on this form of collagen [124]. Hydrolyzed collagen powder is available in the market as a dressing for moderate to heavily exudative wounds. A small randomized clinical trial on patients with burn wounds that used a hydrolyzed collagen supplement suggested a promising role for HC in wound healing [125].

### 6.4. Collagen Bioink

Three dimensional (3D) bioprinting is an evolving adaptive manufacturing technique that offers the development of wound treatments that can deal with the issues presented by traditional wound dressings (i.e., need for frequent dressing change, adherence to wound tissue making dressing changes painful) [126]. The bioprinting process integrates cell-laden hydrogels, also called bioinks, together with motorized systems to create complex structures that can be catered precisely to the patient or situation in question [79,80,126,127,128]. In 2009, a 3D-printed human skin construct incorporating collagen I together with fibroblasts and keratinocytes was the first successful attempt at creating a skin implant. This was followed by other studies that have improved on this attempt and even tested them in situ in a porcine model. The porcine studies showed that the collagen–fibrinogen bioink-printed skin implants incorporating cells significantly enhanced wound re-epithelialization compared to control treatments. Further developments in this line include a laser-assisted bioprinting and robotic solid freeform fabrications to provide contactless, automated solutions using collagen bioinks [129]. As of 2020, there are only about 30 published research articles on this topic.

Collagen bioinks are currently the most popular material for 3D engineering, primarily because of the history of their use in clinical practice, biocompatibility and low immunogenicity. Collagen I is the most common type used for bioink manufacturing and has been used in the laboratory-based bioprinting of skin, bone and cartilage, cardiovascular tissues, liver, nerve regeneration and cornea with limited testing conducted in vitro and in vivo (small animal models) [79,80,126].

A key issue with collagen bioinks is the need for specific pH and temperatures to initiate matrix gelation that could be toxic to cells [130]. Additional challenges include precise tissue detailing (including development of structures like sweat glands and hair follicles) and the need for improved cross-linking techniques for structural control of the bioink-generated product [126]. The production of constructs for large wound areas is a challenge. Furthermore, the search for a 3D printed human skin graft that can support functions such as thermoregulation, touch or sweat is still unfulfilled [129,131].

## 7. Clinical Studies

A search of clinicaltrials.gov using the criteria (collagen + skin + wound) identified 45 clinical studies. This may be viewed as early phase in clinical development. Various formats of collagen dressings have been included in these studies (Table 1). Among the top three formats used in the context of skin health and wound healing are matrices, dietary supplements and sponges with antimicrobials incorporated. Composites of collagen with silk fibroin, alginate and other polymers were also noted. Some of these studies were terminated or withdrawn (~11%) either due to funding limitations, change in study prioritization or undisclosed reasons. Several studies are in the actively recruiting stage. The lack of results associated with the completed studies makes the evaluation of the impact of these collagen applications for clinical use difficult at this time.

## 8. Collagen Wound Dressing Market

In 2019, the global collagen dressings market was valued at approximately USD 926 million and is projected to expand at a compound annual growth rate (CAGR) of ~5% from 2020 to 2030 [132,133]. North America is projected to dominate the global collagen dressings market. Key drivers of the market are collagen composites that include an antimicrobial principle. Despite the rising interest in alternative (and less immunogenic) sources of collagen, bovine origin collagen dominates the markets. Tissue engineering advances such as the electrospinning and 3D bioprinting methods of producing collagen composites are expected to positively impact the market [132,133].

## 9. Closing Remarks

A Pubmed search using the keywords collagen and wound healing lists >10,000 publications promoting various collagen formats as being ideal for wound-healing applications. The poor mechanical and thermal properties of collagen are moving the field towards the use of blends with other materials such as alginate, chitosan and cellulose. However, depending on the ratio of mixtures in these blends, it may not be possible to clearly delineate the exact impact of the collagen component on the treatment outcomes. Most of the published studies are based on in vitro or small animal models. Preclinical porcine studies are necessary to test the translational value of the reported basic science investigations. Collagens can directly modulate the wound microenvironment, serve as a scaffold for cellular attachment and function or deliver biologically active principles or antimicrobials to aid in wound healing. Therefore, for effective translational value, personalization or tailoring collagen biomaterials to the therapeutic need is critical. This nuance is not captured well in current research. While technology is evolving rapidly to produce customized collagen composite scaffolds or nanoparticles incorporating stem cells and other bioactive molecules, the research that can bring these products to clinical practice is limited. The leap from bench to bedside requires rigorous preclinical and clinical testing with demonstrable beneficial outcomes and that is a gap in current collagen biomaterials translational research.

## Figures and Tables

**Table 1:** Collagen dressing formats for wound healing clinical studies.

Collagen Format	Number of Studies
Matrix/scaffold/mesh	15
Dietary supplement	8
Sponge	7
Percutaneous induction/microneedling	5
Composites (e.g., silk fibroin, alginate)	5
Gel	3
Paste/powder	2

## Data Availability

Not applicable.
